# Elevated remnant cholesterol predicts poor outcome in patients with premature acute coronary syndrome: a retrospective, single-center study

**DOI:** 10.1007/s11239-025-03147-6

**Published:** 2025-07-17

**Authors:** Ming Zhang, Jibin Chen, Wei Xia, Zhen Yu, Chunquan Zhang, Wenying Wang, Yibing Shao, Bin Wang

**Affiliations:** 1 School of Clinical Medicine, Shandong Second Medical University, Weifang, China; 2 Department of Cardiology, Qingdao Municipal Hospital, University of Health and Rehabilitation Sciences, No. 5 Donghai Middle Road, Shinan District Qingdao, Qingdao, 266000 China

**Keywords:** Premature acute coronary syndrome, Elevated remnant cholesterol, Outcomes

## Abstract

**Background:**

The association between remnant cholesterol (RC) and recurrent cardiovascular events following acute coronary syndrome (ACS) is well-documented. However, RC-stratified analysis specifically focusing on patients with premature ACS (PACS), defined as initial disease onset occurring at ≤ 55 years of age in men or ≤ 65 years of age in women, remains limited.

**Objectives:**

This study aimed to elucidate the clinical characteristics and subsequent cardiovascular events in patients with PACS, comparing those with high RC levels to those with low RC levels.

**Methods:**

In this retrospective cohort study, 820 PACS patients were consecutively recruited between January 2019 and January 2020. RC was calculated as total cholesterol minus high-density lipoprotein cholesterol minus low-density lipoprotein cholesterol. Patients with RC levels ≥ 66.6 percentile were classified as high RC. The primary endpoint was major adverse cardiovascular and cerebrovascular events (MACCE), including cardiovascular death, myocardial infarction (MI), stroke, ischemia-driven revascularization, or hospitalization for unstable angina or heart failure.

**Results:**

Among the 820 patients enrolled, 277 (33.8%) were classified as high RC and 543 (66.2%) as low RC. The high RC group exhibited a higher prevalence of traditional risk factors, including diabetes (33.6% vs. 27.3%, *p* = 0.04), hypertension (68.2% vs. 61.3%, *p* = 0.04), and hyperlipidemia (43.3% vs. 31.3%, *p* = 0.001). Levels of glucose (*p* < 0.001), hemoglobin A1C (*p* = 0.005), triglyceride (*p* < 0.001), total cholesterol (*p* < 0.001) and LDL-C (*p* = 0.017) were significantly higher in the high RC group, while HDL-C levels were lower (*p* = 0.001). Over 3 years of follow-up, the high RC group had a significantly higher cumulative incidence of MACCE (16.2% vs. 10.9%; adjusted HR 1.68, 95% CI 1.10–2.59; *p* = 0.02). The increased risk was primarily driven by higher rates of hospitalization for unstable angina (12.3% vs. 7.9%; adjusted HR 1.69, 95% CI 1.03–2.75; *p* = 0.03) and composite cardiac events (14.8% vs. 9.8%; adjusted HR 1.75, 95% CI 1.12–2.73; *p* = 0.01).

**Conclusions:**

In hospitalized PACS patients, the cumulative incidence of MACCE was significantly higher in the high RC group compared to the low RC group over a median follow-up of nearly 3 years. The incremental risk in the high RC group was mainly attributable to higher rates of hospitalization for unstable angina and composite cardiac events. Therefore, close attention should be paid to RC levels, and further exploration is warranted.

**Supplementary Information:**

The online version contains supplementary material available at 10.1007/s11239-025-03147-6.

## Introduction

Acute coronary syndrome (ACS), encompassing unstable angina (UA) to myocardial infarction (MI), represents a significant global health challenge [1, 2]. The increasing incidence of ACS in younger populations, defined as premature ACS (PACS) with age cutoffs varying between < 45–55 years, is particularly concerning due to its association with elevated risks of recurrent cardiovascular events and accelerated mortality [3–6]. Identification reliable biomarkers for prognostic stratification in this vulnerable population is crucial for targeted preventive measures and optimized therapeutic interventions.

While low-density lipoprotein cholesterol (LDL-c) has long been the cornerstone of lipid management in ACS patients [7–9], contemporary research highlights significant limitations of this conventional paradigm. Emerging data indicate that non-LDL lipid fractions, not adequately reflected in traditional lipid profiling, exert critical pathophysiological effects on the initiation and progression of atherosclerotic lesions [10]. Notably, remnant cholesterol (RC) has garnered increasing attention as a clinically significant risk determinant for adverse cardiovascular outcomes [11, 12]. RC encompasses atherogenic lipoproteins including intermediate-density lipoproteins (IDL) and very-low-density lipoprotein (VLDL) remnants, which retain cholesterol-rich cores following triglyceride hydrolysis [13].

Accumulating evidence demonstrates that RC exerts multifaceted atherogenic effects, being mechanistically implicated in endothelial dysfunction, oxidative stress-mediated inflammation, and accelerated progression of vulnerable atherosclerotic plaques [14, 15]. Elevated RC concentrations independently predict incident coronary heart disease, persisting as a residual cardiovascular risk factor even among normolipidemic individuals with optimal LDL-c control (LDL-c < 70 mg/dL) [10, 16]. The pathological lipid triad - characterized by concomitant dysregulation of triglyceride-rich lipoproteins, low HDL-c, and small dense LDL particles - is disproportionately prevalent in PACS populations, underscoring the imperative to delineate RC’s specific contributions within this unique metabolic milieu. Therefore, this study aims to elucidate the clinical characteristics and subsequent cardiovascular events in PACS patients with high versus low RC levels.

## Methods

### Study design and participants

The large-scale, retrospective cohort study assessed clinical characteristics and subsequent cardiovascular outcomes of hospitalized PACS patients at Qingdao Municipal Hospital, University of Health and Rehabilitation Sciences (Qingdao, China) between January 2019 and January 2020, with follow-up until December 2023.

**Fig. 1:**
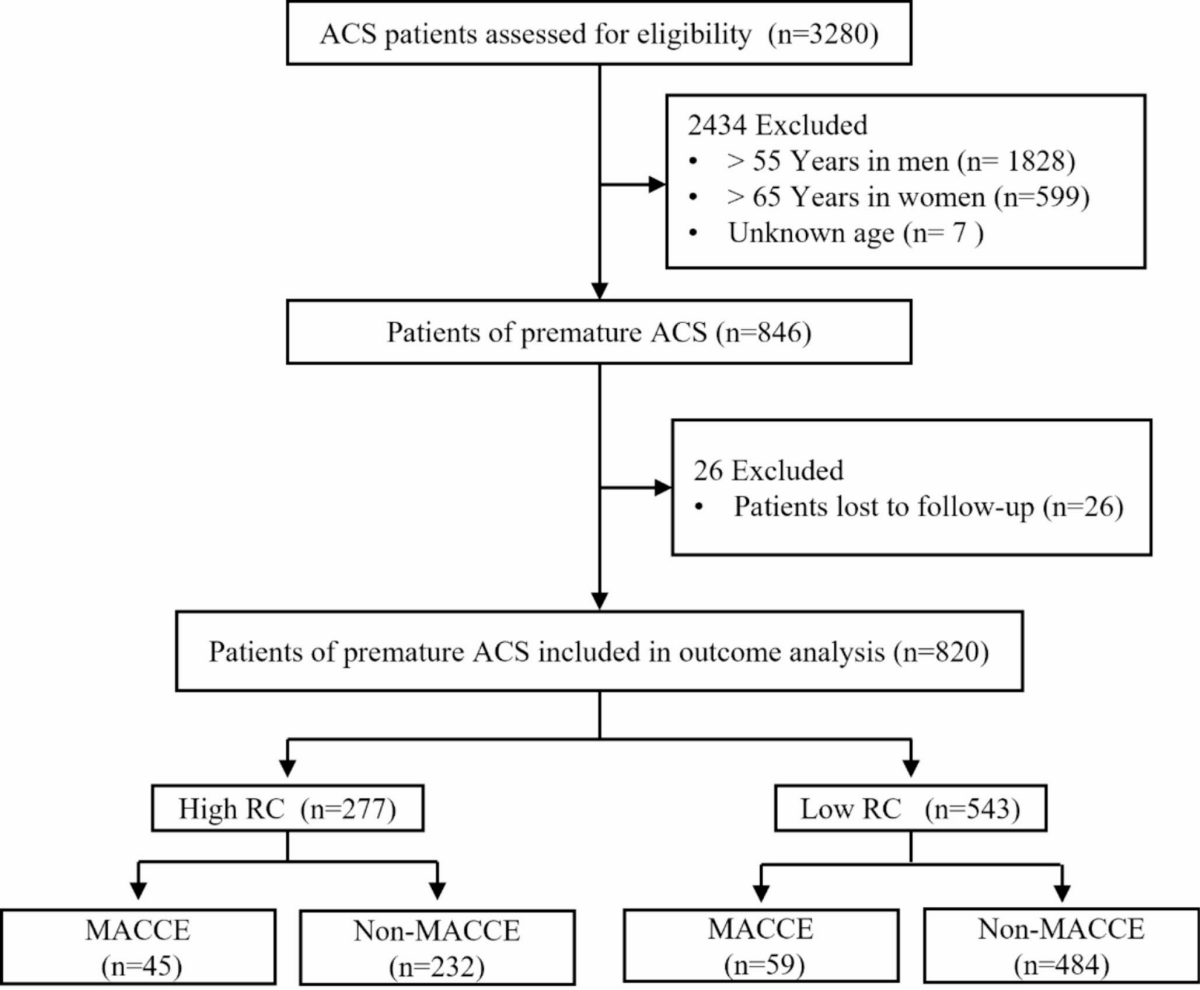
Study flowchart. ACS: acute coronary syndrome, MACCE: major adverse cardiovascular and cerebrovascular event

Patients with ST-elevation myocardial infarction (STEMI) or non-ST-elevation ACS (NSTE-ACS) were included in this study. STEMI was diagnosed based on symptoms characteristic of cardiac ischemia with persistent ST-segment elevation on electrocardiography. NSTE-ACS included non-ST-elevation myocardial infarction (NSTEMI) or unstable angina (UA). NSTEMI was diagnosed based on the presence of persistent ischemic symptoms with elevated cardiac troponin levels but no ST-segment elevation on electrocardiography. UA produced symptoms suggestive of cardiac ischemia without elevated cardiac troponin levels. According to the National Cholesterol Education Program Adult Treatment Panel III guidelines, PACS was defined as initial disease onset occurring at ≤ 55 years of age in men or ≤ 65 years of age in women [17] (Fig. 1).

This study conformed to the Strengthening the Reporting of Observational Studies in Epidemiology guidelines [18], and was conducted in accordance with the amended Declaration of Helsinki [19]. The protocol was approved by the ethics committee of Qingdao Municipal Hospital, University of Health and Rehabilitation Sciences (XS202311001). All patients provided written informed consent.

### Procedures and management

All patients received standard care during index ACS hospitalization, including aspirin, clopidogrel/ticagrelor, low molecular weight heparin, statins, isosorbide mononitrate tablets, angiotensin converting enzyme inhibitors/angiotensin II receptor blocker or metoprolol sustained-release tablets, depending on the heart rate and blood pressure of the individual patient characteristics, according to current guidelines [20]. Experienced senior interventional cardiologists performed the PCI procedures and visual estimation of lesion characteristics. Medical therapies for secondary prevention included dual Antiplatelet therapy (DAPT) at discharge, β-blockers at discharge, statins at discharge, angiotensin converting enzyme inhibitors/angiotensin receptor blockers at discharge, smoking cessation counseling, and cardiac rehabilitation counseling.

### Follow-up and end-points

All patients were followed-up and Clinical events were collected via clinic visit, medical records or telephone calls by research staff blinded to the patients’ clinical characteristics.

The primary endpoint was MACCE, defined as a composite of cardiovascular death, MI, stroke, ischemia-driven revascularization or hospitalization for UA or heart failure. Secondary endpoints included individual components of the primary endpoint, a composite of cardiovascular death, MI or ischemic stroke, a composite of cardiac event, all-cause death and repeat revascularization.

### Statistical analyses

We used counts and percentages (%) to describe qualitative variables and mean ± SD or medians with interquartile ranges (IQRs) for continuous variables. Categorical variables were analyzed by χ2 or Fisher’s exact tests, as appropriate. Continuous variables, where appropriate, were compared by an unpaired t-test or Mann-Whitney U test. Kaplan-Meier survival analysis was performed by log-rank test. Hazard ratios (HR) and 95% confidence interval (95% CI) were calculated with the fully adjusted Cox proportional hazard regression models, which including age, body mass index, smoking, hypertension, diabetes mellitus, hyperlipidemia, prior MI, prior stroke, left ventricular ejection fraction, multi-vessel disease and clinical presentation (acute MI vs. unstable angina). For patient who experienced more than one adverse outcome, only the first adverse event was considered for this analysis. All statistical analyses were calculated by SPSS V.26.0 (IBM SPSS, Armonk, New York, USA). A two-sided *P* < 0.05 was considered statistically significant.

## Results

### Baseline clinical and procedural characteristics

Among 820 patients, 277 (33.8%) were classified as high RC and 543 (66.2%) as low RC. The high RC group had higher prevalence of traditional risk factors, including diabetes (33.6% vs. 27.3%, *p* = 0.04), hypertension (68.2% vs. 61.3%, *p* = 0.04), and hyperlipidemia (43.3% vs. 31.3%, *p* = 0.001). Levels of glucose (*p* < 0.001), hemoglobin A1C (*p* = 0.005), triglyceride (*p* < 0.001), total cholesterol (*p* < 0.001) and LDL-C (*p* = 0.017) were significantly higher in the high RC group, while HDL-C levels were lower(*p* = 0.001). There were no differences in clinical characteristics and discharge medications between the two groups (Tables 1 and 2).

**Table 1:** Baseline clinical characteristics by 2 groups

Subjects	All	High RC	Low RC	*p*-value
	(*n* = 820)	(*n* = 277)	(*n* = 543)	
**Demographics**				
Age, years	49.5 ± 7.7	49.2 ± 8.2	49.6 ± 7.4	0.42
BMI, kg·m^− 2^	27.6 ± 3.8	28.0 ± 3.6	27.4 ± 3.9	0.005
Waist, cm	100 (94–106)	101 (96–107)	98 (92–105)	0.001
Waist-to-hip ratio	0.98 (0.94–1.01)	0.98 (0.95–1.02)	0.97 (0.94-1.00)	0.001
Systolic BP, mm Hg	125 (116–137)	127 (118–137)	125 (116–137)	0.42
Diastolic BP, mm Hg	78 (70–86)	79 (70–88)	77 (70–85)	0.10
**Medical history**				
Diabetes mellitus	241 (29.4)	93 (33.6)	148 (27.3)	0.04
Hypertension	522 (63.7)	189 (68.2)	333 (61.3)	0.04
Hyperlipidemia	290 (35.4)	120 (43.3)	170 (31.3)	0.001
Family history of PCAD	49 (6.0)	23 (8.3)	26 (4.8)	0.04
Prior myocardial infarction	121 (14.8)	41 (14.8)	80 (14.7)	0.97
Prior stroke	58 (7.1)	21 (7.6)	37 (6.8)	0.69
Previous PCI	161 (19.6)	52 (18.8)	109 (20.1)	0.66
Previous CABG	8 (1.0)	3 (1.1)	5 (0.9)	0.82
Smoking				0.57
Yes	405 (49.4)	133 (48.0)	272 (50.1)	
No	415 (50.6)	144 (52.0)	271 (49.9)	
Drinking				0.29
Yes	270 (32.9)	98 (35.4)	172 (31.7)	
No	550 (67.1)	179 (64.6)	371 (68.3)	
**Baseline tests**				
Glucose, mmol/L	5.87 (5.28–7.55)	6.13 (5.35–8.48)	5.76 (5.23–7.05)	<0.001
Hemoglobin A1C, %	6.0 (5.5–6.9)	6.1 (5.6–7.4)	5.9 (5.5–6.7)	0.005
Triglyceride, mmol/L	1.57 (1.14–2.19)	2.45 (1.87–3.27)	1.33 (1.01–1.67)	<0.001
Total Cholesterol, mmol/L	4.13 (3.45–4.91)	4.60 (3.55–5.42)	3.91 (3.26–4.57)	<0.001
HDL-C, mmol/L	0.98 (0.86–1.15)	0.94 (0.83–1.10)	1.01 (0.87–1.17)	0.001
LDL-C, mmol/L	2.45 (1.91–3.17)	2.57 (2.00-3.33)	2.40 (1.83–3.07)	0.017
Hs-CRP, mmol/L	2.13 (0.8–6.5)	2.46 (1.05–6.28)	2.00 (0.69–6.60)	0.13
LVEF, %	62 (58–66)	62 (58–66)	62 (56–65)	0.13

Data are presented as n, mean ± SD, median (interquartile range) or n (%), unless otherwise stated. BMI: body mass index; BP: blood pressure; CABG: coronary artery bypass grafting; HDL-C: high density lipoprotein cholesterol; hs-CRP: high-sensitivity C-reactive protein; LDL-C: low-density lipoprotein cholesterol; LVEF: left ventricular ejection fraction; PCAD: premature coronary artery disease; PCI: percutaneous coronary intervention

**Table 2:** Clinical presentations and management by 2 groups

Subjects	All	High RC	Low RC	*p*-value
	(*n* = 820)	(*n* = 277)	(*n* = 543)	
**Diagnosis**				0.05
STEMI	166 (20.2)	44 (15.9)	122 (22.5)	
NSTEMI	149 (18.2)	59 (21.3)	90 (16.6)	
Unstable angina	505 (61.6)	114 (62.8)	331 (61.0)	
**Procedures**				
Coronary angiography	797 (97.2)	268 (96.8)	529 (97.4)	0.58
LM	50 (6.1)	9 (5.6)	41 (6.2)	0.74
LAD	607 (74.0)	118 (73.3)	489 (74.2)	0.81
LCX	451 (55.0)	79 (49.1)	372 (56.4)	0.09
RCA	431 (52.6)	74 (46.0)	357 (54.2)	0.06
Number of vascular lesions				0.37
0	89 (10.9)	25 (9.0)	64 (11.8)	
1	224 (27.3)	73 (26.4)	151 (27.8)	
≥ 2	507 (61.8)	179 (64.6)	328 (60.4)	
PCI	516 (62.9)	182 (65.7)	334 (61.5)	0.24
CABG	38 (4.6)	11 (4.0)	27 (5.0)	0.52
**Medications on discharge**				
Aspirin	802 (97.8)	270 (97.5)	532 (98.0)	0.64
P_2_Y_12_ inhibitors	746 (91.0)	249 (89.9)	497 (91.5)	0.44
β-blockers	656 (80.0)	224 (80.9)	432 (79.6)	0.66
ACEIs/ARBs	500 (61.0)	176 (63.5)	324 (59.7)	0.28
Statins	806 (98.3)	268 (96.8)	538 (99.1)	0.52

Data are presented as n, median (interquartile range), n (%) or n/N (%), unless otherwise stated

ACEI: angiotensin-converting enzymes inhibitor; ARB: angiotensin receptor blocker; CABG: coronary artery bypass grafting; LAD: left anterior descending artery; LCX: left circumflex artery;

LM: left main artery; NSTEMI: non-ST-segment-elevation myocardial infarction; PCI: percutaneous coronary intervention; RCA: right coronary artery; STEMI: ST-segment-elevation myocardial infarction. The number of vascular lesions = 0 indicates that coronary angiography did not reveal any significant coronary artery stenosis or obstruction, or the stenosis diameter was less than 50%

### Outcomes by RC

**Fig. 2:**
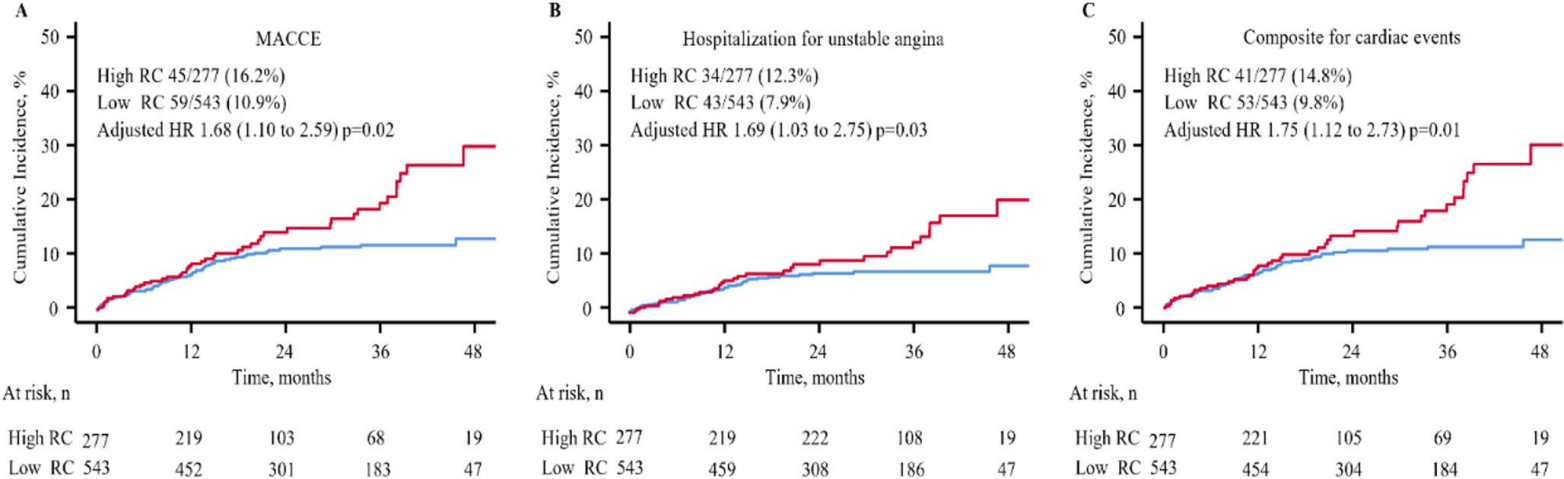
Cumulative incidence of MACCE, Composite for cardiovascular death, MI, or stroke, and Composite for cardiac events by 2 groups. Kaplan-Meier estimates and fully-adjusted HR for cumulative incidence of MACCE **(A)**, Composite for cardiovascular death, MI, or stroke **(B)**, and Composite for cardiac events (includes cardiovascular death, MI, ischemia-driven revascularization or hospitalization for unstable angina or heart failure, **C)** high RC and Low RC. HR: hazard ratio; MACCE: major adverse cardiovascular and cerebrovascular event; MI: myocardial infarction

During 3 years of follow-up, the high RC group, compared with low RC, have a significantly higher cumulative incidence of MACCE (16.2% vs. 10.9%; adjusted HR 1.68, 95% CI 1.10–2.59; *p* = 0.02). The increased risk was primarily driven by higher rates of hospitalization for UA 12.3% vs. 7.9%; adjusted HR 1.69, 95% CI 1.03–2.75; *p* = 0.03) and composite for cardiac events including cardiovascular death, MI, ischemia-driven revascularization or hospitalization for UA or heart failure (14.8% vs. 9.8%; adjusted HR 1.75, 95% CI 1.12–2.73; *p* = 0.01).(Fig. 2, Tables 3 and 4)

**Table 3:** Clinical events up to 3 years by 2 groups

Variables	All	High RC	Low RC	*p*-value
	(*n* = 820)	(*n* = 277)	(*n* = 543)	
**MACCE**	104 (12.7)	45 (16.2)	59 (10.9)	0.029
Cardiovascular death	5 (0.6)	3 (1.1)	2 (0.4)	0.21
Myocardial infarction	13 (1.6)	5 (1.8)	8 (1.5)	0.46
Stroke	12 (1.5)	4 (1.4)	8 (1.5)	0.62
Ischemia-driven revascularization	48 (5.9)	16 (5.8)	32 (5.9)	0.94
Hospitalization for unstable angina	77 (9.4)	34 (12.3)	43 (7.9)	0.031
Hospitalization for heart failure	2 (0.2)	1 (0.4)	1 (0.2)	0.63
**Composite for cardiovascular death**,** MI**,** or ischemic stroke**	30 (3.7)	12 (4.3)	18 (3.3)	0.46
**Composite for cardiac events** ^**#**^	94 (11.5)	41 (14.8)	53 (9.8)	0.032
**Allcause death**	9 (1.1)	4 (1.4)	5 (0.9)	0.50
**All repeat revascularization**	81 (9.9)	31 (11.2)	50 (9.2)	0.37

MACCE: major adverse cardiovascular and cerebrovascular event; MI: myocardial infarction

#: includes cardiovascular death, myocardial infarction, ischemia-driven revascularization or hospitalization for unstable angina or heart failure

**Table 4:** Cox regression analyses evaluating the risk of cardiovascular events in the 2 groups

Variables	Unadjusted		Fully adjusted	
	HR (95% CI)	*p*-value	HR (95% CI)	*p*-value
**MACCE**	1.69 (1.14–2.49)	0.009	1.68 (1.10–2.59)	0.02
Cardiovascular death	3.30 (0.55–19.79)	0.19	5.77 (0.78–42.6)	0.09
Myocardial infarction	1.24 (0.40–3.81)	0.71	1.55 (0.43–5.63)	0.51
Stroke	1.06 (0.32–3.53)	0.92	0.51 (0.10–2.72)	0.43
Ischemia-driven revascularization	1.03 (0.56–1.87)	0.93	1.10 (0.57–2.10)	0.78
Hospitalization for unstable angina	1.72 (1.10–2.70)	0.02	1.69 (1.03–2.75)	0.03
Hospitalization for heart failure^**†**^	2.35 (0.15–37.57)	0.55	-	-
**Composite for cardiovascular death**,** MI**,** or stroke**	0.39 (0.67–2.89)	0.38	1.42 (0.60–3.37)	0.42
**Composite for cardiac events** ^**#**^	1.71 (1.13–2.57)	0.01	1.75 (1.12–2.73)	0.01
**Allcause death**	1.74 (0.47–6.52)	0.41	4.32 (0.86–21.6)	0.08
**All repeat revascularization**	1.27 (0.81–1.99)	0.30	1.23 (0.75–2.02)	0.42

Data are presented as median (IQR). ^#^: includes cardiovascular death, myocardial infarction, ischemia-driven revascularization or hospitalization for unstable angina or heart failure. Model adjusted for age, body mass index, smoking, hypertension, diabetes mellitus, hyperlipidemia, prior myocardial infarction, prior stroke, left ventricular ejection fraction, multi-vessel disease and clinical presentation (acute myocardial infarction vs. unstable angina). ^**†**^: Univariate and/or multivariate Cox regression was not done due to no or few number of events. CI: confidence interval; HR: hazard ratio; MACCE: major adverse cardiovascular and cerebrovascular event

## Discussion

Our study provides novel insights into the prognostic implications of RC in PACS patients, revealing three principal findings: First, elevated RC levels (≥ 66.6th percentile) were independently associated with a 68% increased risk of MACCE during medium-term follow-up. Second, this excess risk predominantly manifested as recurrent unstable angina requiring hospitalization and composite cardiac events. Third, the high-RC phenotype exhibited distinct metabolic derangements characterized by atherogenic dyslipidemia (elevated triglycerides, total cholesterol, and LDL-c with concomitant HDL-c depression), impaired glucose homeostasis, and clustering of traditional cardiovascular risk factors.

The heightened MACCE risk aligns with RC’s pathophysiological role in atherosclerosis progression. RC-rich lipoprotein remnants infiltrate arterial walls, undergoing oxidative modification to trigger endothelial dysfunction and foam cell formation through matrix metalloproteinase activation and fibrous cap destabilization [21, 22]. This mechanism is particularly relevant in PACS populations where non-obstructive coronary disease and microvascular dysfunction prevail, as RC’s small particle size (< 70 nm) facilitates endothelial penetration and promotes ischemia through both macrovascular and microvascular pathways [23, 24]. The pro-inflammatory milieu in high-RC states, evidenced by elevated HbA1c and fasting glucose levels, may exacerbate endothelial progenitor cell dysfunction a finding corroborated by studies linking RC to impaired vascular repair mechanisms in metabolic syndrome populations [22, 24].

Notably, RC demonstrated prognostic discrimination beyond conventional lipid parameters, with 31.3% of low-RC patients meeting hyperlipidemia criteria yet exhibiting better outcomes. This residual risk phenomenon mirrors findings from large cohort studies where RC variability independently predicted metabolic dysfunction-associated steatoses liver disease (MASLD) and carotid intima-media thickening, even among normolipidemic individuals [21, 25]. Our results extend recent Mendelian randomization evidence showing triglyceride-rich lipoproteins mediate cardiovascular risk independent of LDL-c, suggesting RC may serve as a superior therapeutic target in statin-treated PACS patients with residual dyslipidemia [22].

The strong RC-diabetes association (33.6% vs. 27.3%, *p* = 0.04) unveils a bidirectional metabolic cycle: insulin resistance enhances hepatic VLDL secretion while impairing lipoprotein lipase activity, perpetuating RC accumulation - mechanism supported by NHANES data showing RC-HOMA-IR correlations in MAFLD patients (β = 0.17, *p* < 0.001 at RC < 30 mg/dL) [23, 26]. This interplay may explain why high-RC patients exhibited poorer glycemic control despite comparable antidiabetic therapy utilization. Clinically, RC monitoring could identify candidates for intensified therapy, such as fibrates or omega-3 fatty acids, which reduce RC by 25–40% through enhanced lipoprotein lipase activity—a strategy supported by trial data showing RC-lowering interventions reduce recurrent ischemia in metabolic syndrome populations [22, 24]. However, recent studies have underscored that elevated levels of lipoprotein(a) [Lp(a)] are associated with earlier onset and greater complexity of coronary artery disease (CAD) in patients with ACS. Lp(a) is a critical risk factor for early atherogenesis, and aggressive lipid-lowering strategies may be necessary in primary prevention [27]. Notably, while our study focused on RC, it did not specifically measure Lp(a) levels. Therefore, incorporating Lp(a) considerations into future analyses will help to more comprehensively understand the residual cardiovascular risk in patients with metabolic syndrome (such as PACS patients) and optimize therapeutic strategies.

The discordance between LDL-C and RC risk profiles (high-RC/low-LDL-C group: OR 2.04 for hypertension) underscores the need to expand lipid management beyond LDL-centric paradigms [24]. Our findings align with UK Biobank evidence where RC ≥ 1.0 mmol/L increased IHD-T2D multimorbidity risk by 214% (HR 3.14), suggesting RC quantification could refine secondary prevention strategies in young ACS survivors [22]. Future trials should evaluate whether targeting RC thresholds (e.g., < 0.82 mmol/L as proposed in breast cancer cohorts) improves outcomes in this population [26, 28].

Several limitations of our study need to be noted. First, the single-center retrospective design introduces potential selection bias, though mitigated by consecutive enrollment and standardized protocols. Second, compared with direct measurement, using the Fried Ewald formula to calculate RC may underestimate the true level. However, several studies have shown that the calculated residual cholesterol and the actually measured residual cholesterol have similar accuracy in predicting the risk of major adverse cardiovascular events [29, 30]. Third, the absence of granular data on socioeconomic status, physical activity, and dietary habits limits causal inference. Fourth, the observational nature precludes causal inference between RC and outcomes. Fifth, the analysis considers only the first adverse event, potentially underestimating total event burden, especially in high-risk patients. Finally, all patients in the present study were Chinese, so the results should be interpreted and generalized to other ethnic groups with caution since dissimilar metabolic levels exist among different races.

## Conclusions

In hospitalized PACS patients, the cumulative incidence of MACCE was significantly higher in the high RC group compared to the low RC group during a median follow-up of nearly 3 years. The incremental risk in the high RC group was mainly attributable to higher rates of hospitalization for unstable angina and composite cardiac events. Therefore, close attention should be paid to RC levels, and further exploration is warranted to refine lipid management strategies in PACS patients.

## Electronic supplementary material

Below is the link to the electronic supplementary material.

Supplementary Material 1

## Data Availability

No datasets were generated or analysed during the current study.
