# Supplementary material for: Elevated remnant cholesterol predicts poor outcome in patients with premature acute coronary syndrome: a retrospective, single-center study
Source: J Thromb Thrombolysis. 2025 Jul 17;58(7):785–93. doi: 10.1007/s11239-025-03147-6 (PMC12611984; doi:10.1007/s11239-025-03147-6)
Supplement: Supplementary file 1 — Supplementary Material 1 [file 11239_2025_3147_MOESM1_ESM.pptx]

## Slide 1
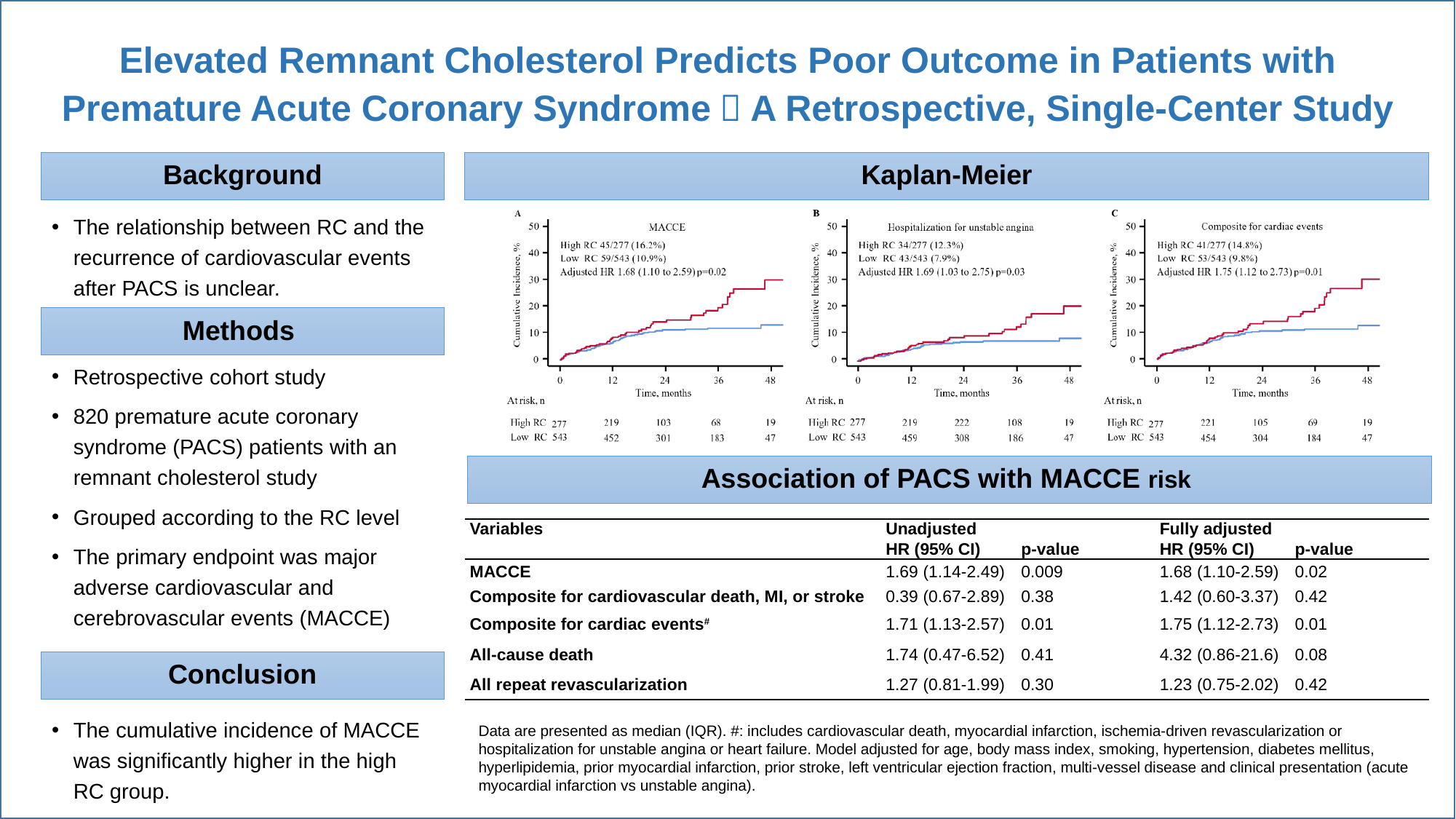

# Elevated Remnant Cholesterol Predicts Poor Outcome in Patients with Premature Acute Coronary Syndrome：A Retrospective, Single-Center Study
Background
Kaplan-Meier
The relationship between RC and the recurrence of cardiovascular events after PACS is unclear.
Methods
Retrospective cohort study
820 premature acute coronary syndrome (PACS) patients with an remnant cholesterol study
Grouped according to the RC level
The primary endpoint was major adverse cardiovascular and cerebrovascular events (MACCE)
Association of PACS with MACCE risk
| Variables | Unadjusted | | Fully adjusted | |
| --- | --- | --- | --- | --- |
| | HR (95% CI) | p-value | HR (95% CI) | p-value |
| MACCE | 1.69 (1.14-2.49) | 0.009 | 1.68 (1.10-2.59) | 0.02 |
| Composite for cardiovascular death, MI, or stroke | 0.39 (0.67-2.89) | 0.38 | 1.42 (0.60-3.37) | 0.42 |
| Composite for cardiac events# | 1.71 (1.13-2.57) | 0.01 | 1.75 (1.12-2.73) | 0.01 |
| All‑cause death | 1.74 (0.47-6.52) | 0.41 | 4.32 (0.86-21.6) | 0.08 |
| All repeat revascularization | 1.27 (0.81-1.99) | 0.30 | 1.23 (0.75-2.02) | 0.42 |
Conclusion
The cumulative incidence of MACCE was significantly higher in the high RC group.
Data are presented as median (IQR). #: includes cardiovascular death, myocardial infarction, ischemia-driven revascularization or hospitalization for unstable angina or heart failure. Model adjusted for age, body mass index, smoking, hypertension, diabetes mellitus, hyperlipidemia, prior myocardial infarction, prior stroke, left ventricular ejection fraction, multi-vessel disease and clinical presentation (acute myocardial infarction vs unstable angina).
